# Prototype Test of Resilient Friction Materials for Seismic Dampers

**DOI:** 10.3390/ma16237336

**Published:** 2023-11-25

**Authors:** Antonella Bianca Francavilla, Massimo Latour, Gianvittorio Rizzano

**Affiliations:** Department of Civil Engineering, University of Salerno, 84084 Fisciano, Italy; mlatour@unisa.it (M.L.); g.rizzano@unisa.it (G.R.)

**Keywords:** intermetallics, friction resistance, devices, damping, mechanical testing

## Abstract

In recent decades, low-yielding seismic devices based on the use of friction dampers have emerged as an excellent solution for the development of building structures with improved reparability and resilience. Achieving an optimal design for such low-yielding seismic devices requires precise control of bolt preloading levels and predictability of the friction coefficient (CoF) between the damper interfaces. While various types of friction devices exist that are capable of providing significant energy dissipation, ongoing research is focused on the development of novel friction materials that exhibit a stable hysteretic response, high CoF values, minimal differences between static and dynamic CoF, and predictable slip resistance. In this context, an experimental campaign was conducted at the STRENGTH Laboratory of the University of Salerno to evaluate the behaviour of new friction shims employing specially developed metal alloys. Specifically, the influence of the characteristics of the contact surfaces in the sliding area on the behaviour and performance of the friction device was analysed. The tests followed the loading protocol recommended by EN12159 for seismic device qualification. Monitored parameters included preloading force values and the evolution of slip resistance. The friction value was determined, along with its degradation over time. Finally, the material’s performance in terms of hysteretic behaviour was assessed, providing a comparison of the tested specimens in terms of slip force degradation and energy dissipation capacity.

## 1. Introduction

Modern seismic codes are designed to meet precise performance goals, ensuring no damage or downtime during standard load combinations (Serviceability Limit States) while allowing controlled damage during rare load combinations caused by severe seismic events (Ultimate Limit States). Within the context of Ultimate Limit State (ULS) design, various options exist to determine the failure mode of a structure, with a primary focus on enhancing structural ductility at both local and global scales within the capacity design framework. In general, a structure’s ability to endure nonlinear deformations depends on inducing damage in predetermined zones and engaging them in a plastic range to absorb the inelastic demand. For steel Moment Resisting Frames (MRFs), following the design procedures outlined in EC8 [1], one approach involves damaging beams using full-strength joints and over-strength columns (continuous frames). Alternatively, a second approach concentrates structural damage in partial strength connections [1,2], with specific detailing rules to ensure that their rotational capacity aligns with the seismic demand (semi-continuous frames). While the design procedures proposed by EC8 have been extensively validated through decades of rigorous research by multiple groups, regardless of the chosen design strategy, the main limitation of conventional approaches lies in the need for structural damage development. Although crucial for preserving structural efficiency and human safety, this damage represents a significant source of both direct and indirect losses during rare seismic events [3,4]. Over past decades, various strategies have been proposed to address this challenge. Among them, supplemental energy dissipation systems have gained significant attention since the 1990s, offering an array of dissipative devices strategically placed in specific zones of the structure that are expected to experience high relative displacements or velocities during severe ground motions [5,6,7,8,9]. Structural damping systems range from passive systems that rely on inherent properties, through semi-active systems with limited adaptability, to active systems that actively and dynamically control damping properties, necessitating external power for optimal performance. The choice depends on the desired level of control, energy considerations, and the specific requirements of the structure [10,11].

According to these systems, it is possible to rely on dissipative dampers explicitly designed for energy dissipation, thereby limiting damage to the main structure, and reducing the seismic inelastic demand. Numerous dissipative devices based on this concept have been proposed, utilizing simple dissipative mechanisms such as metal yielding, dry friction, and fluid viscosity. Furthermore, when considering the advantages of passive dampers, especially friction-based dampers renowned for their cost-effectiveness and simplicity, it becomes apparent that these devices play a crucial role. Despite their simplicity, they effectively contribute to energy dissipation, mitigating seismic forces and reducing potential damage to the main structure. Friction dampers are among the most popular passive control devices due to their simplicity and reliability. The friction damper was initially proposed by Pall et al. in 1980 [12,13]. These dampers dissipate seismic energy effectively through sliding friction to protect the main structure—a method employed globally [14,15]. Mualla et al. [16,17] developed a novel rotational friction damper to dissipate energy and protect buildings during moderate and severe earthquakes. Shaking table tests and numerical simulations by Liao et al. [17] showed that rotational friction dampers could effectively control the lateral displacement and story drift of the test structure under seismic load. Sanati et al. [18] conducted tests on a rotational friction viscoelastic damper to enhance the energy dissipation performance compared to rotational friction dampers.

**Figure 1:**
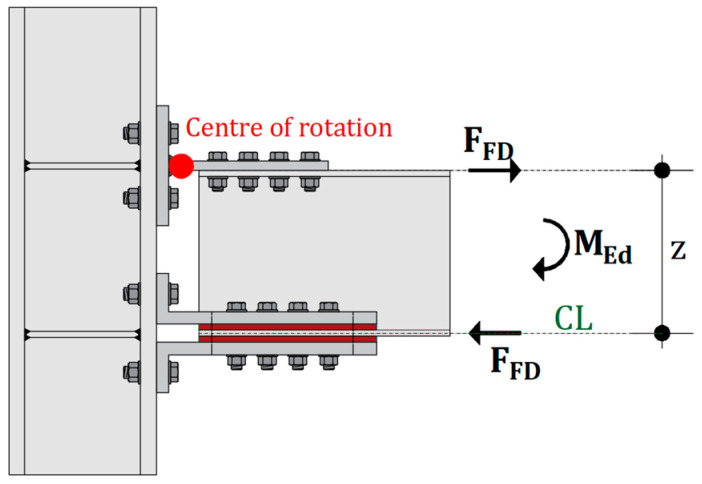
Mechanical scheme of friction connections.

However, even with these supplementary energy dissipation design strategies, the introduction of dissipaters enhances damping, but does not completely eliminate structural damage, as adequate sway displacements of the main structure are still required in order to activate the seismic dissipaters. To address the limitations of conventional design methods, a design approach involving friction connections has been introduced as a solution capable of combining the strengths of the three aforementioned approaches. This method aims to prevent structural damage at both the Serviceability Limit State (SLS) and the Ultimate Limit State (ULS) by incorporating specific types of friction-based damping devices into connections. This design approach allows for the design of rigid frames with fully rigid connections, similar to full-strength design, with a resistance closely aligned with the nominal beam resistance. Additionally, it enables substantial energy dissipation, like supplementary energy dissipation strategies, while simultaneously avoiding structural damage. More recently, friction connections (Figure 1) have been analysed in funded research projects [19,20,21,22,23,24,25,26,27]. In those connections, the bending moment $M_{Ed}$ transmitted from the beam to the column can be controlled by adjusting the slippage force of the friction damper $F_{FD}$ acting in the Center Line (CL) of the connections. This action can be evaluated as the ratio between the bending moment $M_{Ed}$ the and the lever arm z, which is defined as the distance from the mid-thickness of the bottom flange to the upper flange of the beam.

The force $F_{FD}$ is a result of the product of the friction coefficient μ and the number of friction interfaces $n_{s}$, multiplied by the sum of the pre-tightening forces applied with the bolts $F_{p}$. Therefore, precise control of the bolt preloading force and accurate characterization of the friction coefficient of the material used for the friction interface are crucial for controlling the resistance of the friction connections. The preloading force applied by the bolts can be controlled using methods recommended by EN1090-2 [28] (e.g., combined, torque, DTI washers), designed to ensure a minimum of 95% reliability in the tightening specified by the code.

Conversely, characterizing the friction coefficient’s value requires experimental determination and is contingent upon multiple factors. Notably, the friction coefficient of an interface is significantly influenced by the materials used for the friction interface and key tribological properties, such as surface finishing, micro and macro hardness, shear resistance of the materials, and surface roughness. In the last few years, many materials have been investigated and their efficiency in friction connections analysed [29,30]. In fact, in the European Union-funded research project FREEDAM, materials with potential for use in bolted friction dampers were largely studied [23,29,30,31,32,33,34]. Typically, the damper exhibited cyclic behaviour, characterized by a stable response for only a few cycles of sliding. Subsequently, degradation occurred due to the wear of the friction shims and the loss of initial bolt pretension [35]. This aspect was critical because, following a cyclic loading history, the bolts experienced a loss of up to 70% of their initial pretension in the post-sliding phase, significantly deteriorating the material’s initial performance by about 60%. Therefore, despite previous research demonstrating that stable and repeatable hysteretic loops can be achieved with this type of joint for a few cycles of sliding, the degradation of initial bolt pretension and damage to the friction shims resulting from a severe event can jeopardize the structural safety of the building. This is due to a reduction in joint sliding resistance and a decrease in lateral load-bearing capacity during subsequent events. Consequently, although restoring the initial safety of a building structure with friction joints using current technology necessitates the replacement of friction shims and the replacement/re-tightening of bolts, the repair operations are not immediate and present a significant hindrance to the structure’s ability to rapidly restore its previous performance.

In order to address this problem, in this work a specific metal alloy coating has been investigated. The devices realized with this material can exhibit a stable and repeatable hysteretic behaviour with negligible degradation if properly pre-set. To characterize the friction coefficient for the selected material, a preliminary experimental campaign has been conducted at the STRENGTH laboratory (STRuctural ENGineering Testing Hall) of the University of Salerno, following guidelines outlined in EN 1090-2 [28] and EN 15129 [36]. The main outcomes of this experimental activity pertain to static and kinetic friction coefficients, encompassing an assessment of bolt force degradation and effective damping degradation.

## 2. Materials and Methods

Numerous studies available in the literature have highlighted that the proper functioning of friction devices is dependent on *(i) the selection of a friction material with stable hysteretic behaviour, (ii) the initially imposed preload value on the bolts*, and *(iii) the ability to maintain this value nearly unchanged over time* [26,37].

The identification of a stable material with excellent hysteretic performance has been the focus of numerous studies and research projects funded by the European community. This aspect is central in this paper, where the behaviour of a new material, possessing stability characteristics, is analysed, making it well-suited for the design of reversible and resilient steel connections.

Regarding aspect *(ii)*, aiming to minimize preload losses, experimental tests, and statistical variability analyses have been developed, revealing that, for the pertinent applications, the initial preload should vary within a force range of 40–60% of the maximum preload $F_{p,Cd}$ specified by Eurocode 3 [2], as follows:

(1) $$F_{p,Cd}=\frac{0.7A_{s}f_{ub}}{\gamma_{M7}}$$

where $A_{s}$ is the tensile stress area of the bolt, $f_{ub}$ is the ultimate resistance of the bolt, and $\gamma_{M7}=1.1$ is a partial safety factor.

In fact, in ref. [30] it has been pointed out that a reduction of the preloading force, as expected, results in a lower loss of bolt preload and lower energy degradation. However, to ensure that structural performance is upheld, excessively low preload values are either not applicable or are strongly discouraged.

**Figure 2:**
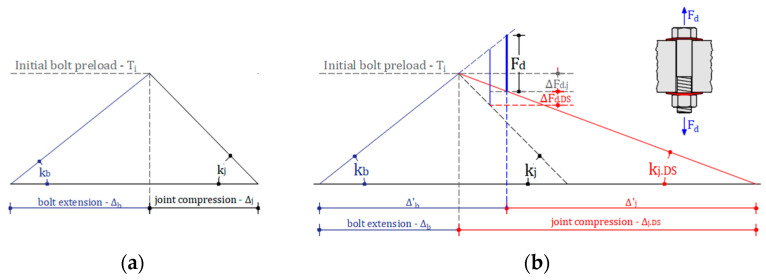
Joint diagram: (**a**) traditional system; (**b**) bolted system with DS subjected to external force.

To address aspect *(iii)*, the use of conical washers in the bolted assemblies of the device was analysed, to enhance the deformability of the assembly and mitigate preload losses in the bolt. In fact, regardless of the loss cause, the capacity of a bolted assembly to preserve the initial clamping force is significantly influenced by the relative stiffness of the bolt and preloaded plies. This concept can be elucidated using the “*joint diagram*”, a graphical representation that combines the force–deflection curves of the various components within the bolted joint (Figure 2a). It provides a comprehensive chart illustrating the equilibrium of the joint in terms of both bolt elongation and joint compression. The joint diagram acts as a valuable tool for designers, facilitating the anticipation of alterations in clamping and bolt forces resulting from external loads imposed on the joint, or relaxation induced by factors such as the deterioration of friction shims. As emphasized in [26,35,37], the Belleville washers provide a spring-like action to compensate for relaxation, settlement, or external loads that may lead to a reduction in clamping force.

The benefit deriving from the use of a more deformable bolting assembly using Belleville conical washers is exemplified in Figure 2b, for the case of a bolting assembly subjected to an external force. As it is possible to easily note from Figure 2b, the higher deformability of the joint results in a lower loss of clamping force.

Therefore, based on past experimental evidence, by considering a combination of a friction material with stable hysteretic behaviour and limiting preload losses over time, it is expected that the connections will exhibit improved resilience and maintain their structural integrity after severe earthquakes. Although this study does not specifically focus on the possible bolting assemblies to be adopted, it is evident that selecting an assembly able to reduce the loss of bolt preload is crucial. Instead, the capability of the selected material to dissipate energy and withstand cyclic loading without significant degradation is preliminarily investigated in the following sections.

## 3. Experimental Tests

### 3.1. Experimental Layout

Following from the considerations outlined in the previous section, the evaluation of material properties is conducted with initial bolt preload values calibrated at 60% of the proof preloading load, with bolting assemblies equipped with custom-designed Belleville disc springs whose geometrical properties are reported in [35].

For the assessment of the coefficient of friction of the analysed materials, the experimental layout was assumed to be similar to that described in [30], where analogous tests were conducted. In particular, the specimens were composed by steel plates assembled to evaluate the uni-axial behaviour of the friction interface. Basically, to this scope, the specimen is connected to the testing machine by means of a slotted plate realized in AISI 304 stainless steel and a steel plate. To these plates, pre-stressed external steel plates and friction pads are bolted by assemblies composed by means of M20 class 10.9 HV bolts and customized Belleville washers manufactured by Solon Manufacturing Co., Chardon, OH, USA.

This configuration has been recognized based on prior research, acknowledging that when interfaces incorporate friction pads with a steel plate, elevated friction coefficients can be attained by pairing materials with a significant contrast in superficial hardness. The distinction in superficial hardness between the plates in contact is pivotal, as hypothesized by Bowden and Tabor [38], linking the friction coefficient (μ) of a metal interface to the ratio of the shear resistance of the weakest material ($\sigma_{0}$) and the superficial hardness of the softest material ($\sigma_{0}$) composing the interface:

(2) $$\mu =\frac{s_{0}}{\sigma_{0}}.$$

Thus, achieving a high friction coefficient necessitates either a high shear resistance of the weakest material, or an exceptionally low superficial hardness of the softest material. In the proposed friction damper for application to resilient friction connections, where the internal surface is constructed from stainless steel AISI 304 (chosen for its corrosion resistance crucial to the durability of the dampers) and characterized by a superficial hardness of approximately 130 HV, the coupled material must exhibit a substantially lower or higher superficial hardness. In this prototype test, the chosen friction material is characterized by a high superficial hardness.

Additionally, to evaluate the influence of the surface treatment of plates at the slippage interface on the friction behaviour and performance, in some specimens the friction shims and/or the slotted stainless steel plate underwent specific surface treatments. In particular, the stainless steel plate was subjected to sandblasting treatment to increase the surface roughness, while the friction shims underwent axial compression to eliminate surface asperities.

**Figure 3:**
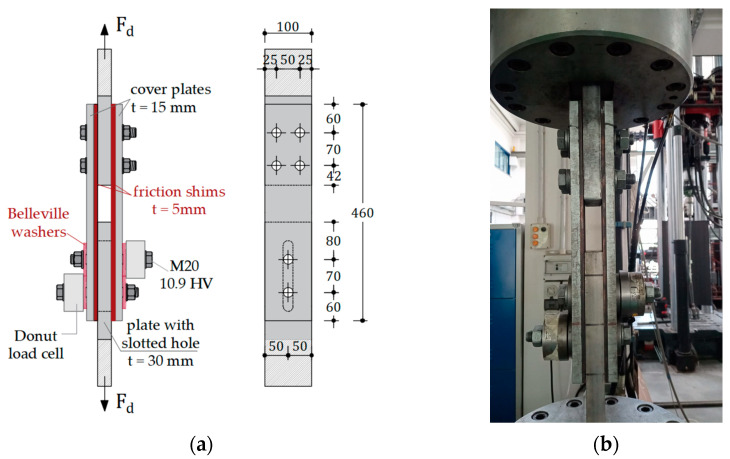
Specimen for slip tests: (**a**) experimental layout; (**b**) specimen during the test.

The typical layout is displayed in Figure 3a. The load protocol was selected in compliance with EN15129 [36]; thus, the specimens underwent testing involving a cyclic loading process carried out in three stages, with the amplitudes progressively increasing to 25%, 50%, and 100% of the device’s maximum design displacement. The initial two stages comprised 5 cycles each, while the third stage consisted of 40 cycles. The maximum amplitude was determined to align with the typical displacements observed at the damper level within a connection, and was set at 25 mm. The testing setup comprised a Schenck Hydropuls S56 universal testing machine, a manual torque wrench, and two Futek LTH500 donut load cells with a maximum capacity of 222 kN. Specifically, the universal machine is equipped with a hydraulic piston capable of a loading capacity of approximately 630 kN, a maximum stroke range of +/− 125 mm, and a self-balanced steel frame designed to counteract axial loads. The axial displacement and slippage force of the device were monitored using transducers and load cells integrated into the testing machine. Additionally, the variation of the preload force applied to bolts was tracked utilizing the Futek LTH500 donut load cells positioned at the lower bolts (Figure 3b).

As mentioned previously, in order to assess the influence of friction interface surfaces on the hysteretic behaviour of the friction material, specimens were identified by varying the surface characteristics of the stainless steel plate and/or the friction shims. To this end, 4 tests were conducted with different specimens.

The first test was conducted strictly following the layout proposed in [36] on specimen FM-1. Subsequently, the bolts of the device were preloaded to achieve the specified initial preload force (FM-2), and the test was repeated. To assess the influence of the surface properties of the stainless steel plate in the contact zone on the slippage section, for the FM-3 specimen a sandblasted plate replaced the original stainless steel slotted plate. Finally, a device equipped with pre-squashed friction shims (FM-4) was tested. In particular, the shims are subjected to compressive forces using the Schenck Hydropuls S56 universal machine to uniformly eliminate surface irregularities.

In all the analysed scenarios, the bolting assemblies were equipped with custom-preset Belleville washers, and the initial bolt preload was set at 60% of the proof value $F_{p,Cd}$. To achieve the target preload, the force was applied to the bolts with a hand torque wrench and monitored by means of annular load cells. An amplification factor of the nominal torque equal to 1.1 was adopted to match the minimum preload with 95% reliability, as suggested by EN 1090-2. A summarized overview of the test parameters is provided in Table 1.

### 3.2. Test Results

**Figure 4:**
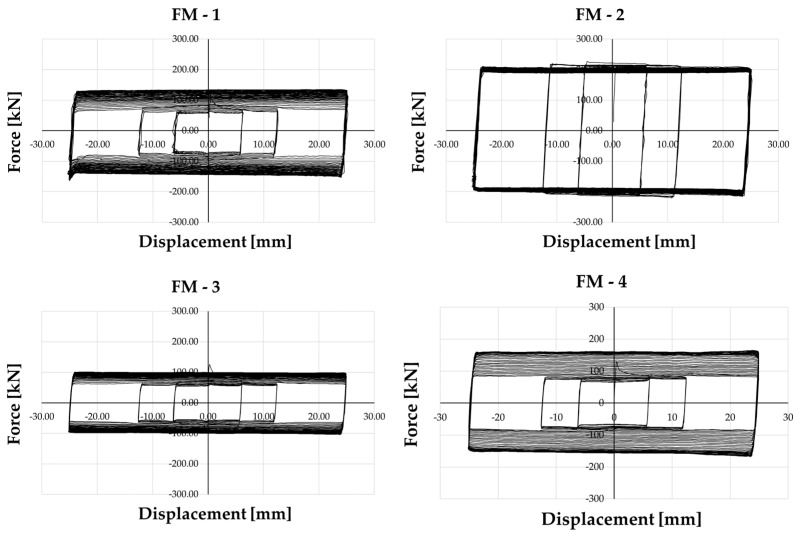
Force—Displacement curves.

To assess the performance under cyclic loading conditions, the force and displacements applied to the devices are monitored, as well as the variation of the bolt preload force. In Figure 4, the force–displacement curves of the four specimens are depicted. In the case of FM-1, the cyclic response displayed an initial slip force of approximately 115 kN, which significantly increased as the sliding phase commenced, reaching a force value of about 65 kN. Subsequently, a new increase in resistance is observed until, with the increase in cumulative displacement, it reaches an almost constant value, equal to about 135 kN and then upwards of the initial slip force. Considering the force achieved during the occurrence of the first slippage, the force increase at the end of the test is about 110%.

During the repeated FM-2 test, conducted after repeating the preloading procedure on the bolt within the sliding part of the device FM-1, the force–displacement relationship trend was quite different. In fact, an initial slip force of about 220 kN was noted. However, upon initiation of the slip phase, a minor force degradation was observed, reaching a force value of about 190 kN. This degradation—approximately 15%—remained nearly constant until the test’s end. This reduction can be attributed to the elimination of surface asperities and the establishment of a more cohesive interface between the two friction surfaces during the previous test.

Differently from the FM-2 test but similar to the FM-1 test response, in the case of the sandblasted slotted plate (FM-3), the initial slip force was approximately 126 kN, which drastically decreased as the sliding phase commenced, reaching a force value of about 55 kN. After this significant degradation, the slip force gradually increased, attaining a force value of approximately 100 kN. A response similar to that exhibited by the FM-1 specimen was provided by the FM-4 test, where conditioned friction shims were employed. The initial slip force was around 130 kN, decreasing to approximately 65 kN during the initial cycles until achieving approximately 160 kN at the test’s end.

The force improvement observed at the conclusion of the test, with respect to the force achieved in the occurrence of the first slippage, is about 80% and 145% for specimens FM-3 and FM-4, respectively.

Additionally, in order to conduct a comparative analysis of the friction coefficient across various assemblies, the values of slippage force and bolt forces measured using the donut load cells were utilized. Based on the data obtained from the testing devices, two distinct values for the friction coefficient were identified: an ‘*effective*’ value and an ‘*actual*’ value.

The first value $\mu_{eff}$ of the friction coefficient was computed as the ratio of the slippage force to the sum of the nominal preloading forces applied by the bolts (i.e., 4 × 113.5 kN). This effective value represents the friction coefficient suitable for seismic design, including degradation of the friction coefficient due to surface damage in contact, as well as degradation resulting from bolt loosening. Conversely, the ‘*actual*’ value of the friction coefficient ($\mu_{act}$) was determined as the ratio of the slippage force to the sum of the bolt forces measured by the load cells during the test. The actual value provides a true measure of the friction coefficient, capturing degradation solely attributed to surface damage in contact, while effects arising from bolt loosening are directly measured using the donut load cells.

**Figure 5:**
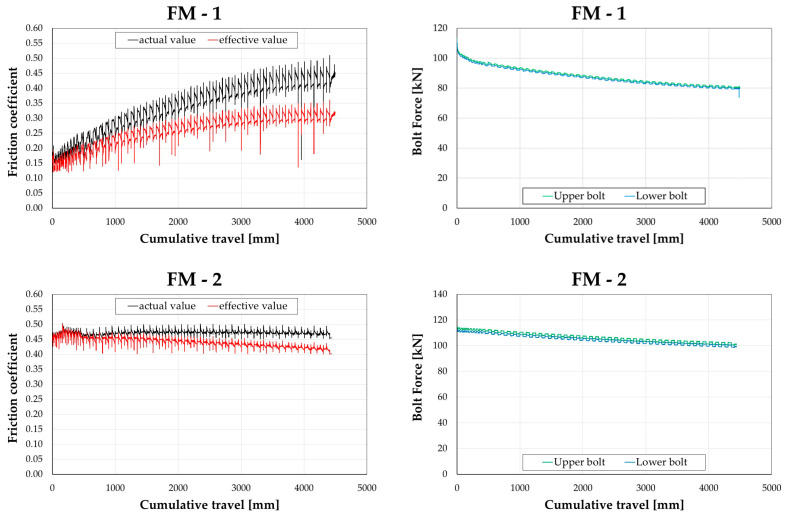

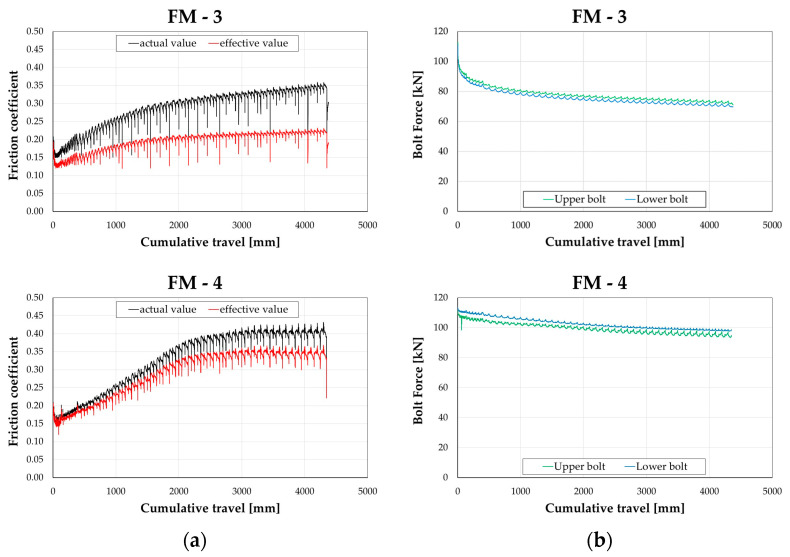
Experimental results: (**a**) friction coefficients versus cumulative travel; (**b**) bolt force versus cumulative travel.

Figure 5 presents diagrams correlating bolt forces (monitored via load cells) and friction coefficients with the cumulative travel of the damper for specimens. The diagrams highlight that both bolts, initially preloaded to attain a load of 113.5 kN, exhibited a gradual reduction in tension throughout the test, culminating in an overall loss of approximately 30%, 11%, 37%, and 15% in the cases of FM-1, FM-2, FM-3, and FM-4, respectively. In all cases—except for FM-2, where the test is a repetition following the retightening of bolts from the previous test—the effective material friction coefficient demonstrates strain-hardening behaviour. In the case of FM-2, there is a slight reduction in the friction coefficient. Analysing the behaviour of the actual friction coefficient allows for an assessment of the material’s performance independent of variations in bolt preloading, solely reflecting the material degradation at the sliding interface. The trend closely follows that described in terms of the effective value, apart from the FM-2 case, where a nearly constant value is observed.

In Table 2, the values of the actual and effective friction coefficients for all the specimens, evaluated at the initial and final stages of the tests, have been reported.

## 4. Discussion

**Figure 6:**
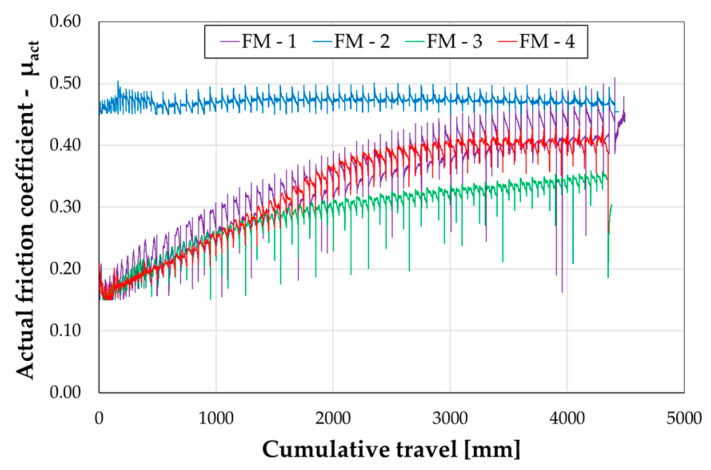
Comparison between tests: actual friction coefficient vs. cumulative travel.

To aid in assessing the impact of the surface treatment of contact surfaces in the sliding zone, Figure 6 depicts the curves of the actual friction coefficient versus cumulative displacement for all the analysed cases. The comparison clearly reveals that surface treatments, such as sandblasting the friction plate or pre-squashing the friction shims, are not particularly effective. In fact, the response of specimens FM-3 and FM-4 does not deviate significantly from that of specimen FM-1. Conversely, conducting the test on a previously tested device yields significantly improved results. It appears that, after eliminating surface asperities, the friction plate plastically deforms the surface over which it slides, removes material, and establishes a coupling that results in stable friction behaviour.

Ultimately, based on the results obtained from the comparison depicted in Figure 6, it can be affirmed that the coupling arising at the sliding interface proves particularly effective only when it forms through the execution of the test. Preliminary surface treatments do not appear to initiate this mechanism, and consequently, they do not augment friction performance.

Obviously, this also has an impact on the ability of bolted assemblies to maintain the initially applied preload. Indeed, the reduction in the thickness of the plates connected by the bolts due to material consumption leads to inevitable preload losses. Since this consumption occurs exclusively in the initial phase of testing, in the case of an FM-2 test or a repeated test the loss of preload is minimal.

Finally, combining stable behaviour of the material friction coefficient and lower loss of bolt preload, the FM-2 specimen exhibits a more stable hysteretic behaviour. This makes it the only configuration among those analysed that can be employed in resilient structures with high seismic performance.

In conclusion, the effective damping degradation has been determined following the procedure described in [36]. This parameter primarily indicates a decrease in the energy dissipation capacity of the frictional device. The EN 15129 standards set a 15% limit for this parameter for seismic devices that depend on displacement.

**Figure 7:**
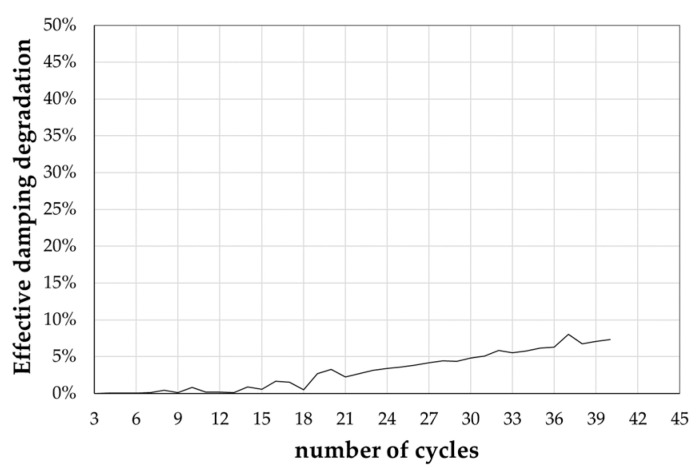
Effective damping degradation.

This parameter cannot be determined for the specimens FM-1, FM-3, and FM-4, as they exhibit non-degrading but instead hardening behaviour. Such behaviour renders them unsuitable for use in seismic and resilient structures, as an increase in strength with increasing cumulative displacement exposes them to structural damage. On the contrary, specimen FM-2 displays an effective damping degradation of approximately 7%, making it suitable for use in low-displacement seismic devices. Figure 7 illustrates the trend of effective damping degradation concerning the number of cycles for specimen FM-2.

## 5. Conclusions

The selection of friction materials with stable hysteresis behaviour for use in friction-based damping devices in seismic-resistant structures is of paramount importance. This paper presents the results of prototype testing of resilient friction materials for seismic dampers, focusing on various friction interfaces created by combining slotted plates and friction shims, along with the influence of specific surface treatments like sandblasting and pre-squashing in the sliding zone.

Preliminary experimental tests suggest that, irrespective of the surface treatment method applied to the contact surfaces in the sliding zone, there is no significant improvement in the performance of friction-based damping devices. Conversely, when testing an unproven device, hysteric behaviour is significantly more stable, exhibiting a resistance degradation of less than 15%. This behaviour is attributed to a combination of a friction coefficient with a nearly constant value throughout the entire test and minimal preload losses in the bolts, equal to about 15%. This improvement is attributed to the development of a more cohesive interface between the two friction surfaces during prior testing.

This conclusion is supported by a comprehensive analysis of hysteresis behaviour under various test configurations, including examination of individual parameters such as effective and actual friction coefficients and effective damper degradation. In fact, both effective and actual friction coefficients at the end of the test exhibit a value that has tripled compared to the value recorded at the beginning of the test. This is the case for devices FM-1, FM-3, and FM-4. In contrast, for device FM-2, the trend of the effective friction coefficient is slightly degrading (less than 10%), while the actual friction coefficient remains practically constant.

Moreover, previously tested devices exhibit nearly constant resistance with increasing cumulative displacement, maintaining a minimal effective damping degradation rate of just 7%. Significantly, this degradation rate falls below the established minimum standard for anti-seismic devices, making these devices well-suited for application in resilient seismic dampers.

## Figures and Tables

**Table 1:** Tests matrix.

Specimen Code	Plate with Slotted Hole	Initial Preload Force ${1.1\cdot 60\%\cdot F}_{p,Cd}$	Friction Shims	Test Type
		[kN]		
FM-1	Stainless steel	113.5 113.5	New	New test
FM-2				Repetition of FM-1
FM-3	Sandblasted Stainless steel	113.5	New	New test
FM-4	Stainless steel	113.5	Pre-squashed	New test

**Table 2:** Experimental results.

Specimen Code	Actual Friction Coefficient		Effective Friction Coefficient	
	Initial Value	Final Value	Initial Value	Final Value
FM-1	0.15	0.45	0.12	0.32
FM-2	0.45	0.45	0.45	0.40
FM-3	0.16	0.30	0.15	0.19
FM-4	0.16	0.28	0.16	0.24

## Data Availability

Data available on request.
